# Shaker/Kv1 potassium channel SHK-1 protects against pathogen infection and oxidative stress in *C. elegans*

**DOI:** 10.1371/journal.pgen.1011554

**Published:** 2025-02-06

**Authors:** Longjun Pu, Jing Wang, Lars Nilsson, Lina Zhao, Chloe Williams, Guanqiao Chi, Jonathan D Gilthorpe, Simon Tuck, Johan Henriksson, Yi-Quan Tang, Sun Nyunt Wai, Changchun Chen

**Affiliations:** 1 Department of Molecular Biology, Umeå University, Umeå, Sweden; 2 Umeå Centre for Molecular Medicine, Umeå University, Umeå, Sweden; 3 Wallenberg Centre for Molecular Medicine, Umeå University, Umeå, Sweden; 4 Department of Medical and Translational Biology, Umeå University, Umeå, Sweden; 5 Institutes of Brain Science, Department of Orthodontics, State Key Laboratory of Medical Neurobiology, MOE Frontiers Center for Brain Science, Shanghai Stomatological Hospital & School of Stomatology, Fudan University, Shanghai, China; 6 Umeå Centre for Microbial Research (UCMR), Umeå University, Umeå, Sweden; 7 Integrated Science Lab (Icelab), Umeå University, Umeå, Sweden; 8 The Laboratory for Molecular Infection Medicine Sweden (MIMS), Umeå University, Umeå, Sweden; University of California San Diego, UNITED STATES OF AMERICA

## Abstract

The Shaker/Kv1 subfamily of voltage-gated potassium (K^+^) channels is essential for modulating membrane excitability. Their loss results in prolonged depolarization and excessive calcium influx. These channels have also been implicated in a variety of other cellular processes, but the underlying mechanisms remain poorly understood. Through comprehensive screening of K^+^ channel mutants in *C. elegans*, we discovered that *shk-1* mutants are highly susceptible to bacterial pathogen infection and oxidative stress. This vulnerability is associated with reduced glycogen levels and substantial mitochondrial dysfunction, including decreased ATP production and dysregulated mitochondrial membrane potential under stress conditions. SHK-1 is predominantly expressed and functions in body wall muscle to maintain glycogen storage and mitochondrial homeostasis. RNA-sequencing data reveal that *shk-1* mutants have decreased expression of a set of cation-transporting ATPases (CATP), which are crucial for maintaining electrochemical gradients. Intriguingly, overexpressing *catp-3*, but not other *catp* genes, restores the depolarization of mitochondrial membrane potential under stress and enhances stress tolerance in *shk-1* mutants. This finding suggests that increased *catp-3* levels may help restore electrochemical gradients disrupted by *shk-1* deficiency, thereby rescuing the phenotypes observed in *shk-1* mutants. Overall, our findings highlight a critical role for SHK-1 in maintaining stress tolerance by regulating glycogen storage, mitochondrial homeostasis, and gene expression. They also provide insights into how Shaker/Kv1 channels participate in a broad range of cellular processes.

## Author summary

Exposure to environmental stimuli, such as bacterial pathogens, increases the production of reactive oxygen species (ROS), leading to oxidative stress and widespread cellular damage. Cells employ various defense mechanisms to counteract ROS-induced damages and maintain cellular homeostasis. In this study, we found that the Shaker/Kv1 channel SHK-1, typically known for its regulation of membrane excitability, also helps protect *C. elegans* against bacterial infections and oxidative stress. Animals lacking *shk-1* are highly sensitive to pathogens and prooxidant exposure, and show defects in gene expression, glycogen storage, and mitochondrial function. Notably, *shk-1* mutants have reduced expression of *catp-3*, encoding one of cation-transporting ATPases that are critical for maintaining ion balance. Restoring *catp-3* expression rescues the defects observed in *shk-1*-deficient animals, suggesting that the loss of SHK-1 likely disturbs ion homeostasis, which can be mitigated by increased levels of *catp-3*. These findings offer a new perspective on the fundamental mechanism by which SHK-1 regulates diverse cellular functions. Moreover, our observations may have implications for how Kv1.3 is involved in various physiological processes in mammals.

## Introduction

Organisms are constantly exposed to a diverse array of environmental and systemic stressors, including temperature fluctuations, variable nutrient availability, pathogen exposure, and toxin encounters. Such exposures typically result in increased production of reactive oxygen species (ROS). When ROS generation overwhelms the capacity of antioxidant systems, it leads to oxidative stress, causing damage to DNA, lipids, and proteins [1,2]. Throughout evolution, animals have evolved intricate, conserved, and finely tuned mechanisms to detect, respond to, and adapt to oxidative challenges. These defensive strategies typically engage a spectrum of evolutionarily conserved cellular processes and signaling pathways, which are essential for maintaining cellular homeostasis, ensuring survival, and promoting resilience under adverse conditions [3].

The interplay between ion channels and ROS has been extensively documented [2,4–6]. Ion channels contain sulfhydryl groups within redox sensitive cysteine and methionine residues, making them susceptible to ROS targeting. Redox modification of ion channels may induce changes in their structures and properties, leading to the alterations in various signaling cascades and downstream physiological responses [6]. Conversely, alterations in ion channel functions can potentially disrupt cellular ion homeostasis and antioxidant mechanisms, resulting in aberrant cellular responses to oxidative stress. Despite the functional significance of the interaction between ion channels and oxidative stress, the dynamic nature of ROS and the intricate regulation of ion channels present substantial challenges in fully understanding detailed aspects of this relationship.

In this study, we aimed to elucidate the molecular mechanisms by which a Shaker/Kv1 subfamily of voltage-gate K^+^ channel, SHK-1/Kv1, regulates the defense against bacterial infection and oxidative stress in *C. elegans*. Mammalian Shaker/Kv1 channels have well-characterized functions in the central nervous system, where they play crucial roles in the repolarization of action potential [7,8]. This group of Kv channels has also been implicated in various non-canonical processes such as cell proliferation, adhesion, and apoptosis [9]. A notable example is Kv1.3, one of the Shaker/Kv1 channels in humans, which was initially discovered in human T cells and subsequently observed in various tissues including brain, skeletal muscles, liver, and kidney [10–12]. It participates in diverse cellular processes, including the control of body weight and insulin sensitivity [13,14].

SHK-1, the sole Shaker/Kv1 channel in *C. elegans*, has been shown to regulate the delayed outward current in muscle cells and the termination of action potential in neurons [15–19]. Loss of SHK-1 channels results in prolonged action potentials and excessive calcium (Ca^2+^) influx [17,18,20]. However, it remains largely unexplored if SHK-1 has roles beyond its regulation of ion flux across membrane. In this study, we show that *shk-1* mutants exhibit pleiotropic phenotypes, including reduced glycogen storage, impaired mitochondrial function, and significant changes in gene expression, which collectively weaken animals’ defense mechanisms against pathogen infection and oxidative stress. In particular, overexpressing *catp-3*, a gene encoding a cation-transporting ATPase, rescued the defects observed in *shk-1* mutants. Given the critical role of SHK-1 in modulating ion flux and the importance of cation transporting ATPases in maintaining electrochemical gradients [17,18,20,21], we propose that the defects associated with *shk-1* mutants are likely caused by prolonged Ca^2+^ influx and subsequent disruption of Ca^2+^-dependent signaling pathways. Overexpression of *catp-3* may restore cellular ion gradients, thereby mitigating the defects in *shk-1* mutants. These findings suggest a potential mechanism by which SHK-1 participates in multiple cellular processes.

## Results

### *shk-1* mutants are susceptible to bacterial pathogens

**Fig 1:**
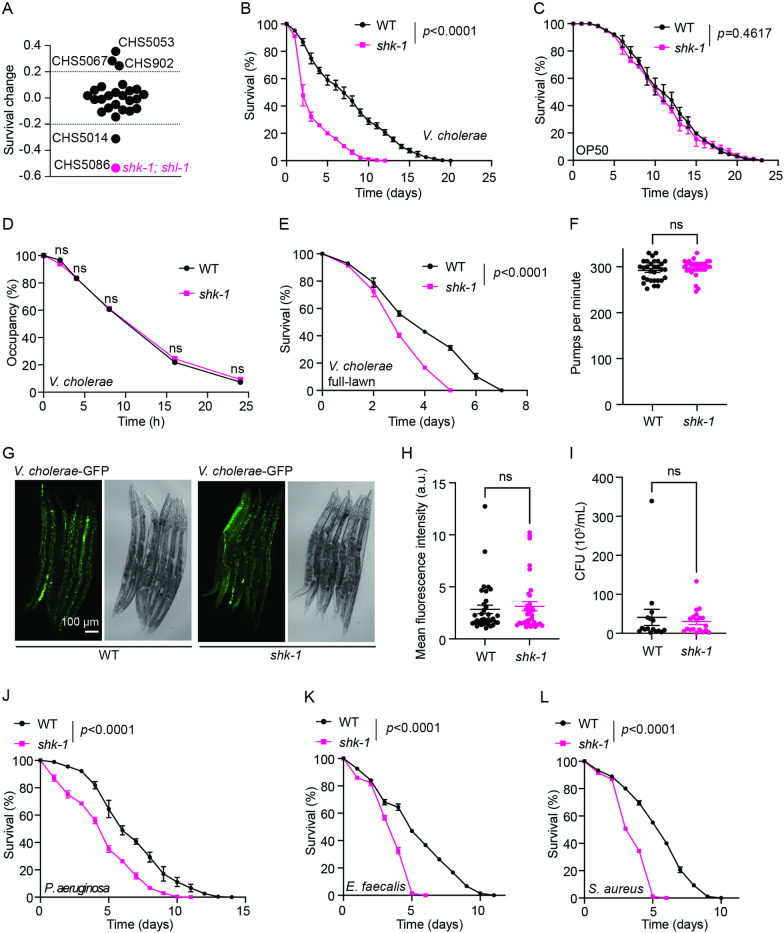
Disruption of *shk-1* increases susceptibility to bacterial pathogens. **(A)** Violin plot of mean survival changes of K^+^ channel mutants relative to WT. The hypersensitive mutant CHS5086 (*shk-1; shl-1*) was marked in magenta. **(B)** Survival of WT and *shk-1(yum1003)* animals upon exposure to *V. cholerae* A1552. n = 3 biological replicates. *p* value is displayed in the plot. log-rank test. **(C)** Lifespan of WT and *shk-1(yum1003)* animals on *E. coli* OP50 at room temperature. n = 3 biological replicates. *p* value is displayed in the plot. log-rank test. **(D)** The percentage of WT and *shk-1(yum1003)* animals remaining on the *V. cholerae* A1552 lawn at different time points. n = 3 biological replicates. ns = not significant. Two-tailed *t* test. **(E)** Survival of WT and *shk-1(yum1003)* animals on the full lawn of *V. cholerae* A1552. n = 3 biological replicates. *p* value is displayed in the plot. log-rank test. **(F)** Pharyngeal pumping per minute of WT and *shk-1(yum1003)* mutants. n = 30 for each strain. ns = not significant. Two-tailed *t* test. **(G)** Representative micrographs showing the accumulation of GFP signals in animals fed with GFP labeled *V. cholerae* A1552 for 16 hours. **(H)** Quantitative analysis of fluorescence intensity in (G). n = 34 for WT and n = 35 for *shk-1(yum1003)* mutants. ns = not significant. Two-tailed *t* test. **(I)** Quantification of colony forming units (CFU) of GFP labeled *V. cholerae* A1552 in WT and *shk-1(yum1003)* mutants upon 16-hour exposure. ns = not significant. Two-tailed *t* test. **(J–L)** Survival of WT and *shk-1(yum1003)* animals upon exposure to *P. aeruginosa* PA14 (J), *E. faecalis* OG1RF (K), or *S. aureus* NCTC8325 (L). n = 3 biological replicates. *p* values are displayed in the plots. log-rank test.

Several K^+^ channels have been implicated in adaptive and innate immune responses in mammals [4]. This prompted us to explore if any K^+^ channels in *C. elegans* contribute to defense mechanisms against infections. In our previous studies on the role of K^+^ channels in acute hypoxia sensing, we disrupted all 72 K^+^ channel-encoding genes across 26 strains (S1 Table) [22]. When exposed to the Gram-negative bacterial pathogen strain *Vibrio cholerae* A1552, several K^+^ mutants exhibited altered susceptibility to the infection (Figs 1A, S1A–S1F, and S1 Table). In particular, *shk-1; shl-1* double mutants were highly susceptible to *V. cholerae* exposure (Figs 1A, S1A, S1G, and S1 Table). When examining the response of each single mutant, we found that the sensitivity of *shk-1* single mutants, but not that of *shl-1* strains, was comparable to that of *shk-1; shl-1* double mutants (Figs 1B and S1G). Additionally, no significant differences in survival between wild type and *shk-1* mutants were observed when animals were fed with either standard laboratory food source OP50 or heat-killed *V. cholerae* (Figs 1C and S1H). These observations suggest that disrupting *shk-1* potentially compromises the defense capacity against living bacterial pathogen without affecting longevity.

Next, we sought to explore the possible causes underlying the enhanced susceptibility to pathogen infection in *shk-1* mutants. *C. elegans* escapes pathogen exposure as a cost-effective survival strategy [23]. Defects in pathogen avoidance often correlate with increased pathogen uptake and enhanced susceptibility. We found that *shk-1* mutants effectively escaped the pathogen at a level comparable to that of wild type animals (Fig 1D). When assaying animals on a full bacterial lawn, which eliminated the effect of pathogen avoidance, *shk-1* mutants were still hypersensitive to *V. cholerae* infection (Fig 1E). In addition, *shk-1* mutants had normal pharyngeal pumping and bacterial colonization in the intestine (Fig 1F–1I). These findings suggest that the enhanced susceptibility to pathogen infection in *shk-1* mutants is unlikely to be caused by defects in pathogen avoidance or increased pathogen uptake.

MakA is a cytotoxin secreted by *V. cholerae* that plays a major role in the killing of *C. elegans* [24–26]. When the *makA* operon from *V. cholerae* was introduced into the non-pathogenic *E. coli* strain Top10, this bacterium failed to repel both wild type and *shk-1* mutants (S1I Fig), but was pathogenic to *C. elegans* (S1J Fig). These observations suggest that distinct factors in *V. cholerae* mediate the killing and repulsion of animals, and further confirm that behavioral avoidance is unlikely to underlie the different susceptibility to bacterial infection between wild type and *shk-1* mutants. We next sought to determine if SHK-1 was specifically required for animals’ tolerance to *V. cholerae* infection. Examining a set of bacterial pathogens revealed that *shk-1* mutants were not only susceptible to the other Gram-negative pathogens such as *Pseudomonas aeruginosa* PA14, but also to the Gram-positive bacteria *Enterococcus faecalis* and *Staphylococcus aureus* (Fig 1J–1L). These findings suggest a broad requirement of SHK-1 in defense against bacterial infection.

### *V. cholerae* infection reprograms the transcriptome in *C. elegans*

**Fig 2:**
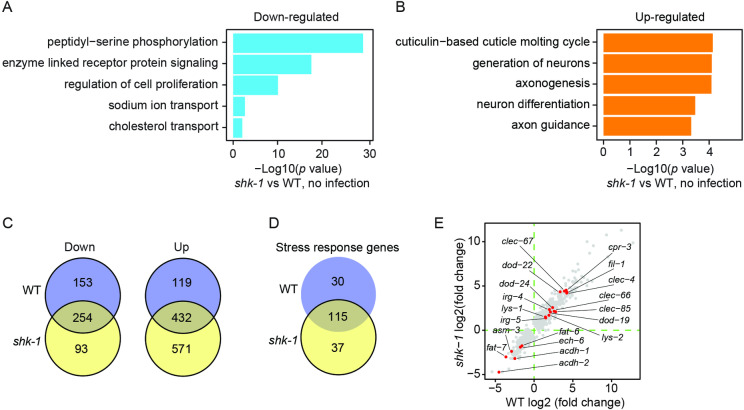
*V. cholerae* exposure substantially remodels gene expression. **(A and B)** GO categories for down- (A) and up- (B) regulated genes in *shk-1(yum1003)* mutants under standard conditions with adjusted *p* <1e-20. **(C)** Venn diagrams displaying significantly down-(left) and up-(right) regulated genes in WT and *shk-1(yum1003)* mutants after 8 hours of exposure to *V. cholerae* A1552. **(D)** Venn diagram showing the number of differentially expressed genes in the GO category ‘stress response’ in WT and *shk-1(yum1003)* mutants after 8 hours of *V. cholerae* infection, with adjusted *p* <1e-20. **(E)** Scatter plot showing the expression fold changes of differentially expressed genes with adjusted *p* value <1e-20 in WT and *shk-1* mutants after 8 hours of exposure to *V. cholerae* A1552. The genes known to be regulated by *P. aeruginosa* PA14 infection were highlighted in red.

Previous studies have shown that disrupting *shk-1* results in prolonged repolarization of membrane potential, resulting in excessive and sustained Ca^2+^ influx [17,18,20]. This could affect various Ca^2+^ dependent signaling pathways, leading to changes in gene expression. Consistent with this, 373 genes were significantly upregulated, and 993 genes were substantially downregulated in *shk-1* mutants compared to the wild type under standard conditions (S2 Table; *p. adj* <1e-20). Gene Ontology (GO) analysis revealed an enrichment of downregulated genes in categories such as peptidyl-serine phosphorylation and sodium ion transport, while upregulated genes were enriched in categories like cuticle molting cycle and neuron generation in *shk-1* mutants (Fig 2A and 2B; *p. adj* <1e-20). Interestingly, the GO term analysis did not indicate significant enrichment of differentially expressed genes in categories related to stress responses, including defense mechanisms against bacterium, in *shk-1* mutants. This suggests that disrupting *shk-1* did not markedly alter the expression of defense response genes under basal conditions.

To investigate if SHK-1 is required for the induced expression of bacterial defense-related genes, we conducted RNA-seq analysis on both wild type and *shk-1* mutant animals following 8 hours of *V. cholerae* exposure. The bacterial infection triggered a dramatic transcriptional response, with 958 and 1350 genes differentially expressed in wild-type and mutant animals, respectively (*p. adj* <1e-20). Both upregulated and downregulated genes showed a substantial overlap between wild type and *shk-1* mutants (Fig 2C). GO term analysis revealed the highest enrichment of upregulated genes in the category of bacterial defense mechanism, while downregulated genes were primarily enriched in the categories of fatty acid metabolic processes in both strains (S2A and S2B Fig; *p. adj* <1e-20). Analysis of genes within the GO category of stress response, which includes those involved in bacterial defense and other stress responses, revealed that 115 out of the 182 differentially expressed genes were shared between wild type and *shk-1* mutants (Fig 2D). The majority of these genes exhibited comparable levels of expression changes (S2C Fig). Moreover, a set of genes significantly regulated by PA14 also exhibited substantial expression changes following *V. cholerae* exposure in both strains (Fig 2E). The fold changes of these genes were similar between the two strains (Fig 2E). These observations suggest that *V. cholerae* infection elicits robust defense responses in both wild type and *shk-1* mutant animals.

### SHK-1 regulates oxidative stress response

**Fig 3:**
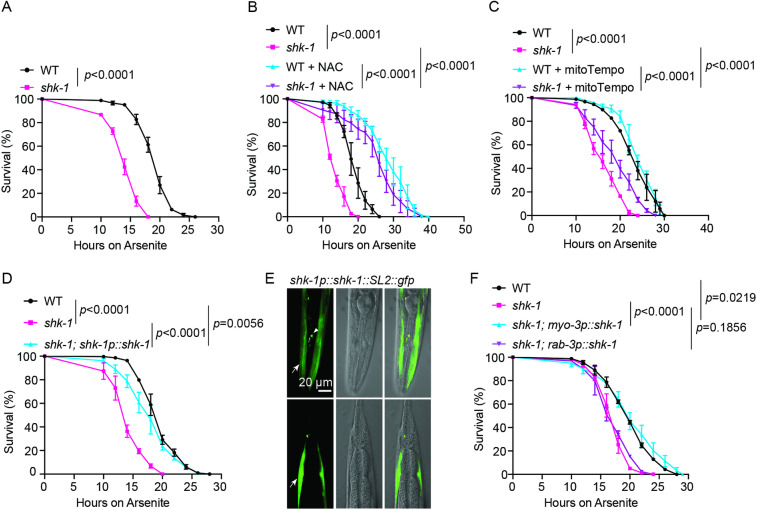
*shk-1* mutants exhibit attenuated tolerance to oxidative stress. **(A)** Survival of WT and *shk-1(yum1003)* animals upon exposure to 7.7 mM arsenite. n = 3 biological replicates. *p* value is displayed in the plot. log-rank test. **(B)** Survival of WT and *shk-1(yum1003)* mutants, with or without the treatment of the antioxidant N-acetylcysteine (NAC), upon exposure to 7.7 mM arsenite. n = 3 biological replicates. *p* values are displayed in the plot. log-rank test. **(C)** Survival of WT and *shk-1(yum1003)* mutants, with or without the treatment of the antioxidant mitoTempo, upon exposure to 7.7 mM arsenite. n = 3 biological replicates. *p* values are displayed in the plot. log-rank test. **(D)** Survival of WT, *shk-1(yum1003)*, and transgenic *shk-1(yum1003)* animals expressing *shk-1* isoform c cDNA under its own promoter upon exposure to 7.7 mM arsenite. n = 3 biological replicates. *p* values are displayed in the plot. log-rank test. **(E)** Representative image showing GFP expression from a *shk-1p::shk-1::SL2::gfp* polycistronic construct. The anterior part (head) is at the top, and the posterior part (tail) is at the bottom. The arrows indicate the body wall muscles and the arrow heads indicate the head neurons. **(F)** Survival of WT, *shk-1(yum1003)* and transgenic *shk-1(yum1003)* animals expressing *shk-1* isoform c cDNA under *myo-3* promoter (muscle) and *rab-3* promoter (neuron) upon exposure to 7.7 mM arsenite. n = 3 biological replicates. *p* values are displayed in the plot. log-rank test.

Pathogen exposure stimulates the production of reactive oxygen species (ROS), leading to oxidative stress in *C. elegans* [27–33]. Hence, we explored if SHK-1 plays a role in defending against oxidative challenges. Our analysis revealed that *shk-1* mutants displayed increased sensitivity to pro-oxidants arsenite and paraquat (Figs 3A, S3A and S3B). This observation was validated using a second null allele of *shk-1* (S3A and S3B Fig). Treating animals with antioxidants N-acetylcysteine (NAC) or MitoTempo significantly improved the survival of *shk-1* mutants upon arsenite exposure (Fig 3B and 3C), supporting a potential role of SHK-1 in defense against oxidative stress. Since the response to oxidative stress is mainly mediated by SKN-1, the *C. elegans* ortholog of mammalian NRF2 [34], it was not surprising that introducing gain-of-function alleles of *skn-1* restored the arsenite tolerance in *shk-1* mutants (S3C and S3D Fig).

*C. elegans* genome contains multiple splicing isoforms of *shk-1* (S3E Fig). To investigate if any specific isoform(s) play a predominant role in response to oxidative stress, we utilized CRISPR/Cas9 to disrupt different subsets of *shk-1* isoforms and subsequently assessed the arsenite sensitivity of mutant animals. It appeared that arsenite susceptibility was only observed in the mutants with all isoforms disrupted (S3F Fig), suggesting that individual isoforms act redundantly to protect animals against oxidative stress. Expressing *shk-1.c* cDNA from its endogenous promoter effectively rescued the defect of *shk-1* mutants (Fig 3D). A transcriptional *gfp* reporter driven by the *shk-1.c* promoter showed that *shk-1* was predominantly expressed in body-wall muscles, as well as in a set of head neurons (Fig 3E). Surprisingly, we did not observe clear fluorescent signals in the body-wall muscles when SHK-1 was endogenously tagged with GFP using CRISPR/Cas9. The GFP signal was clearly detected in IL2L and IL2R neurons, with weaker expression observed in other head neurons (S3G Fig). To determine the tissue in which SHK-1 acts, we selectively expressed *shk-1.c* cDNA in body wall muscles and neurons. We found that expressing *shk-1.c* cDNA specifically in body wall muscles, but not in neurons, rescued the defects of *shk-1* mutants in response to arsenite or *V. cholerae* exposure (Figs 3F and S3H). Furthermore, restoring RNAi-mediated knockdown of *shk-1* expression specifically in body wall muscles of *rde-1* strain, which is deficient in RNAi, was sufficient to render animals susceptible to arsenite (S3I Fig). These data suggest that SHK-1 functions in body wall muscle to modulate the response to oxidative stress and pathogen exposure.

### Impaired glycogen storage compromises oxidative stress tolerance in *shk-1* mutants

**Fig 4:**
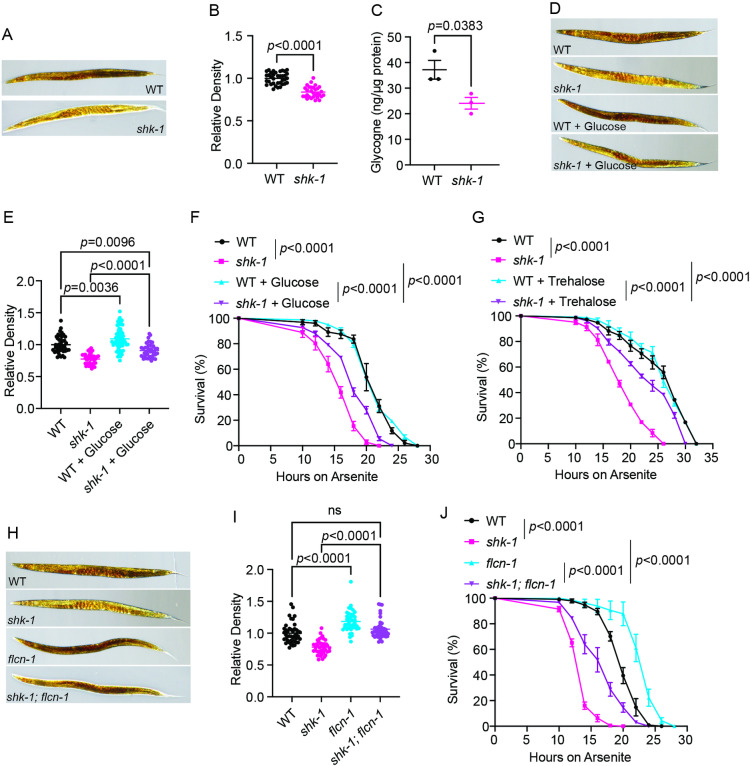
*shk-1* mutants display decreased glycogen storage. **(A)** Representative images showing glycogen staining with iodine in WT and *shk-1(yum1003)* animals. **(B)** Quantitation of the relative optical density in (A). n = 36 (WT) and n = 30 (*shk-1*). *p* value is displayed in the plot. Two-tailed *t* test. **(C)** Glycogen content in WT and *shk-1(yum1003)* animals. The amount of glycogen was normalized to the total protein. n = 3 biological replicates. *p* value is displayed in the plot. Two-tailed *t* test. **(D)** Representative images showing glycogen staining by iodine in WT and *shk-1(yum1003)*, with or without the treatment of 2% glucose. **(E)** Quantitative analysis of the relative optical density in (D). n = 44 (WT), n = 41 (*shk-1*), n = 60 (2% glucose-treated WT), and n = 40 (2% glucose-treated *shk-1*), *p* values are displayed in the plot. ANOVA, Tukey’s multiple comparison. **(F)** Survival of WT and *shk-1(yum1003)*, with or without the treatment of 2% glucose, upon exposure to 7.7 mM arsenite. n = 3 biological replicates. *p* values are displayed in the plot. log-rank test. **(G)** Survival of WT and *shk-1(yum1003)*, with or without the treatment of 5 mM trehalose, upon exposure to 7.7 mM arsenite. n = 3 biological replicates. *p* values are displayed in the plot. Log-rank test. **(H)** Representative images showing glycogen staining with iodine in WT, *shk-1(yum1003), flcn-1(yum1020)*, and *shk-1(yum1003); flcn-1(yum1020)* double mutants. **(I)** Quantitative analysis of the relative optical density in (H). n = 43 (WT), n = 41 (*shk-1*), n = 41 (*flcn-1*), and n = 42 (*shk-1; flcn-1*). *p* values are displayed in the plot, and ns = not significant. ANOVA, Tukey’s multiple comparison. **(J)** Survival of WT, *shk-1(yum1003), flcn-1(yum1020)*, and *shk-1(yum1003); flcn-1(yum1020)* animals upon exposure to 7.7 mM arsenite. n = 3 biological replicates. *p* values are displayed in the plot. log-rank test.

We next aimed to explore the mechanisms underlying the protective role of SHK-1 against bacterial infection and oxidative stress. The importance of Kv1.3, the human ortholog of *shk-1*, in energy metabolism prompted us to speculate that impaired energy reserves or dynamics might account for the compromised stress tolerance in *shk-1* deficient animals [13,14]. We discovered that *shk-1* mutants had significantly reduced levels of glycogen, the primary storage form of glucose (Fig 4A–4C). Notably, previous studies have highlighted the crucial role of glycogen storage in cellular responses to diverse stress conditions. For example, accumulated glycogen can be quickly mobilized to release glucose, supporting the reduction of disulfide bonds and ROS scavenging in both worms and humans [35]. Additionally, glycogen is broken down to generate osmolytes, facilitating a fast defense against hyperosmotic stress [36]. Given the correlation between an organism’s stress tolerance and the level of glycogen storage, we sought to determine if decreased glycogen reserves could be an underlying mechanism for the compromised stress response observed in *shk-1* mutants. We found that glucose supplementation, which partially restored the glycogen levels (Fig 4D and 4E), significantly improved the arsenite tolerance in *shk-1* mutant animals (Fig 4F). Furthermore, the supplementation of trehalose, a disaccharide sugar, also suppressed the arsenite hypersensitivity of *shk-1* mutants (Fig 4G).

To further confirm that increasing glycogen levels could protect *shk-1* mutants against oxidative stress, we examined if mutations associated with elevated glycogen content could suppress the defects observed in *shk-1* mutants. *flcn-1* is the *C. elegans* ortholog of the human *FLCN* gene, which encodes the tumor suppressor Folliculin that plays an evolutionarily conserved role in regulating glycogen storage [37]. Loss of *flcn-1/FLCN* substantially increases glycogen accumulation via constitutive activation of AMP-activated protein kinase [36]. We found that disrupting *flcn-1* restored the glycogen storage in *shk-1* mutants (Fig 4H and 4I), and rescued the susceptibility of *shk-1* mutants to arsenite or *V. cholerae* exposure (Figs 4J and S3J). Collectively, these observations suggest that impaired glycogen accumulation contributes to the defective response to oxidative stress in *shk-1* mutants.

### SHK-1 is required for the maintenance of mitochondrial function

**Fig 5:**
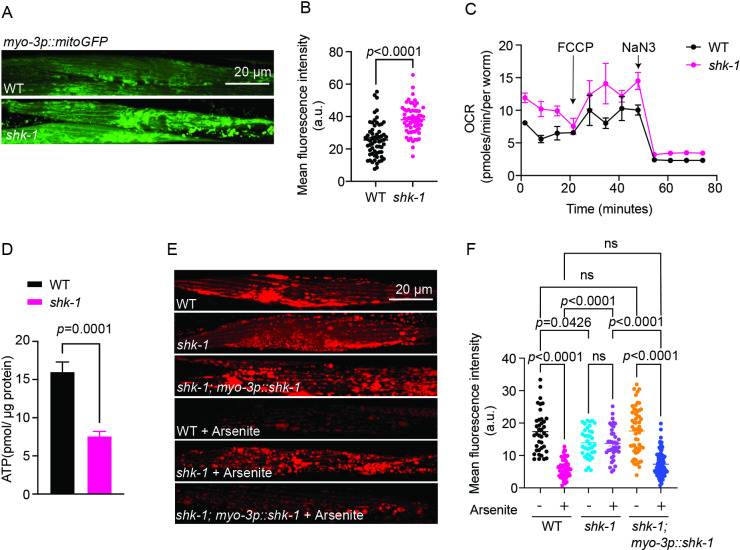
*shk-1* mutants exhibit defects in mitochondrial homeostasis. **(A)** Representative images showing mitoGFP signal in body wall muscle of WT and *shk-1(yum1003)* animals. **(B)** Quantitative analysis of fluorescence intensity in (A). n = 62 for each strain. *p* value is displayed in the plot. Two-tailed *t* test. **(C)** Seahorse analysis of the O_2_ consumption rate (OCR) in WT and *shk-1(yum1003)* animals. Arrows indicate the addition of 100 μM carbonyl cyanide 4-(trifluoromethoxy) phenyl-hydrazone (FCCP) and 400 mM sodium azide (NaN_3_). n = 3 biological replicates. **(D)** Quantification of ATP content in WT and *shk-1(yum1003)* animals. n = 3 biological replicates, each with two technical replicates. *p* value is displayed in the plot. Two-tailed *t* test. **(E)** Representative images showing TMRE staining of body wall muscle in WT, *shk-1(yum1003)*, transgenic *shk-1(yum1003)* expressing *shk-1* isoform c cDNA under *myo-3* promoter, treated with or without 7.7 mM arsenite exposure. **(F)** Quantitative analysis of fluorescence intensity in (E). n = 41 (WT), n = 48 (*shk-1*), n = 56 (*shk-1; myo-3p::shk-1*), n = 49 (WT, arsenite), n = 42 (*shk-1*, arsenite) and n=92 (*shk-1; myo-3p::shk-1*, arsenite). *p* values are displayed in the plot, and ns = not significant. ANOVA, Tukey’s multiple comparison.

Glycogen serves as a rapid source of glucose to fuel ATP production in mitochondria, especially under high-energy-demanding situations. Deficiencies in glycogen storage are accompanied with significant mitochondrial dysfunction, as evidenced by previous studies on patients with glycogen storage disorders [38–40]. We therefore wondered if the reduced glycogen levels in *shk-1* mutants were also associated with any mitochondrial defects. Examining the mitochondrial network in the body-wall muscle using mitochondria-targeted GFP reporter (mitoGFP) revealed a significant increase in mitoGFP intensity in *shk-1* mutants (Fig 5A and 5B). This increase might reflect a compensatory mechanism for the attenuated mitochondrial function. Even though O_2_ consumption rate (OCR) was higher, ATP production was significantly reduced in *shk-1* mutants (Fig 5C and 5D), suggesting possible mitochondrial uncoupling. When assessing mitochondrial membrane potential (ΔΨm) using tetramethylrhodamine, ethyl ester (TMRE), a cell-permeable dye readily absorbed by active mitochondria with high ΔΨm, a substantial decrease in TMRE uptake by mitochondria was observed in *shk-1* mutant animals, suggesting partial ΔΨm dissipation under normal conditions (Fig 5E and 5F). Mild ΔΨm dissipation is often linked to elevated mitochondrial Ca^2+^ entry under stress or pathological conditions [41], suggesting that mitochondrial Ca^2+^ influx may occur in *shk-1* mutants. In addition, arsenite exposure induced a clear depolarization of ΔΨm in wild type animals, but this effect was not observed in *shk-1* mutants (Fig 5E and 5F). The defect of ΔΨm depolarization in response to arsenite was rescued by expressing *shk-1* specifically in the body wall muscle (Fig 5E and 5F). These findings demonstrate that SHK-1 is critical for the maintenance of mitochondrial function and integrity in *C. elegans*.

### A cation-transport ATPase CATP-3 is involved in SHK-1 mediated response to oxidative stress

Our RNA-seq analysis showed that *zip-8* and *cav-1* were among the most differentially expressed genes in *shk-1* mutants (S4A Fig and S2A Table). We wondered if the increased expression of these genes contributes to the mutant phenotypes. In particular, Shaker/Kv1.3 has been reported to interact with caveolin Cav1 in mammals to control apoptosis [42]. The RNAi-mediated knockdown of *zip-8* expression or the disruption of *cav-1* gene did not affect the animals’ response to arsenite, nor did they suppress the arsenite hypersensitivity of *shk-1* mutants (S4B and S4C Fig). In addition, overexpressing these two genes in the wild-type background did not alter animals’ response to arsenite (S4D and S4E Fig). These data suggest that SHK-1 unlikely acts through ZIP-8 and CAV-1 to modulate the response to oxidative stress.

**Fig 6:**
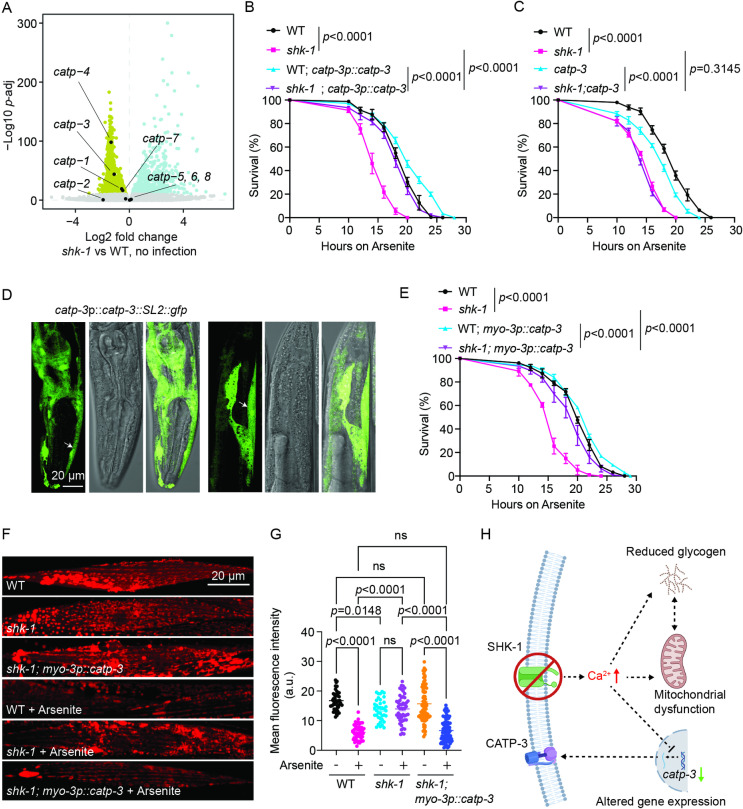
Decreased expression of *catp-3* underlies compromised tolerance to oxidative stress in *shk-1* mutants. **(A)** Volcano plot highlighting the expression of *catp* genes in *shk-1* (*yum1003*) mutants relative to WT without the exposure to *V. cholerae* A1552. **(B)** Survival of WT, *shk-1(yum1003),* transgenic WT and *shk-1(yum1003)* overexpressing *catp-3* genomic DNA under its own promoter upon exposure to 7.7 mM arsenite. n = 3 biological replicates. *p* values are displayed in the plot. log-rank test. **(C)** Survival of WT, *shk-1(yum1003), catp-3(yum1092)* and *shk-1(yum1003); catp-3(yum1092)* double mutants upon exposure to 7.7 mM arsenite. n = 3 biological replicates. *p* values are displayed in the plot. log-rank test. **(D)** Representative images showing GFP expression from a *catp-3p::catp-3::SL2::gfp* polycistronic construct. The anterior part (head) is to the left, and the posterior part (tail) is to the right. **(E)** Survival of WT, *shk-1(yum1003),* transgenic WT and *shk-1(yum1003)* expressing *catp-3* genomic DNA under *myo-3* promoter upon exposure to 7.7 mM arsenite. n = 3 biological replicates. *p* values are displayed in the plot. log-rank test. **(F)** Representative images showing TMRE staining of the mitochondria in body wall muscles in WT, *shk-1(yum1003)*, transgenic *shk-1(yum1003)* expressing *catp-3* genomic DNA under *myo-3* promoter, with or without the exposure to 7.7 mM arsenite. **(G)** Quantitative analysis of fluorescence intensity in (F). n = 43 (WT), n = 46 (*shk-1*), n = 74 (*shk-1; myo-3p::catp-3*), n = 51 (WT, arsenite), n = 49 (*shk-1*, arsenite), and n=88 (*shk-1; myo-3p::catp-3*, arsenite). *p* values are displayed in the plot, and ns = not significant. ANOVA, Tukey’s multiple comparison. **(H)** Graphic representation illustrating a plausible mechanism by which the disruption of *shk-1* impairs stress responses. In *shk-1* mutants, prolonged Ca^2+^ influx disrupts various Ca^2+^ dependent signaling pathways, compromising the animals’ stress tolerance. This was created in BioRender - Chi, G. (2024) https://BioRender.com/f70o631.

GO term analysis revealed an enrichment of differentially expressed genes in the process of sodium ion homeostasis in *shk-1* mutants. Within this category were a set of genes encoding cation-transporting ATPases, including *catp-1*, *catp-3*, *catp-4*, and *catp-7*, known to play critical roles in driving ion movement across membrane and maintaining electrochemical gradients [43]. The expression of these genes was down-regulated by disrupting *shk-1* (Fig 6A). To explore the potential contribution of decreased expression of these genes to the phenotypes observed in *shk-1* mutants, we began by examining if any *catp* mutants exhibited defects in response to oxidative stress. We found that several *catp* mutants, including *catp-2*, *catp-3*, and *catp-6* strains, exhibited enhanced susceptibility to arsenite exposure (S5A–S5E Fig). Consistent with this, *catp-3* mutants were recently shown to be sensitive to cisplatin induced ROS production [44]. Interestingly, increased expression of *catp-3*, but not the other *catp* genes, restored arsenite tolerance in *shk-1* mutants to levels comparable to those observed in the wild type (Figs 6B and S5F–5I). Similar observations were made with *V. cholerae* exposure: *catp-3* mutants were hypersensitive to the bacterial infection (S5J Fig), and overexpressing *catp-3* genomic DNA under its endogenous promoter rescued the defects of *shk-1* mutants in response to *V. cholerae* (S5K Fig). Furthermore, *catp-3; shk-1* double mutants did not exhibit an enhanced sensitivity when compared  to that of *shk-1* single mutants (Fig 6C), implying that CATP-3 and SHK-1 likely act in the same genetic pathway. A transcriptional *gfp* reporter driven by the *catp-3* promoter showed the fluorescent signals primarily in the body wall and pharyngeal muscles in *C. elegans* (Fig 6D), which overlapped with the expression of *shk-1* (Fig 3E). Overexpressing *catp-3* exclusively in body wall muscle significantly improved arsenite and pathogen tolerance of *shk-1* mutants (Figs 6E and S5L). In addition, increased expression of *catp-3* successfully restored the depolarization of mitochondrial membrane potential evoked by oxidative stress in *shk-1* mutants (Fig 6F and 6G). Therefore, we conclude that the decreased expression of *catp-3* gene contributes to the impaired response of *shk-1* mutants to oxidative stress as well as bacterial pathogen exposure. Considering the importance of both SHK-1 and cation-transporting ATPases in regulating ion movement across membranes, these observations also suggest that the defects observed in *shk-1* mutants are likely caused by disrupted ion flux and gradients (Fig 6H), which are mitigated by increased *catp-3* expression.

## Discussion

The Shaker/Kv1 subfamily of voltage-gate K^+^ channels is highly diverse and widely distributed across various tissues, contributing not only to the modulation of excitability in neurons but also to the maintenance of cellular homeostasis in different cell types [7,9]. This ability to regulate cellular homeostasis becomes particularly critical under high energy demanding conditions. In this study, we identify that the *C. elegans* Shaker/Kv1 channel, SHK-1, plays a key role in orchestrating animals’ response to bacterial pathogen infection and oxidative stress. Our findings reveal that disruption of *shk-1* results in altered gene expression, decreased glycogen storage, and disrupted mitochondrial function, compromising animals’ tolerance to stress.

As a voltage-gated K^+^ channel, SHK-1 regulates the outward flow of K^+^ ions to restore the membrane potential after depolarization in neurons and muscle cells [15–18,20]. Its disruption prolongs the repolarization phase, resulting in increased intracellular Ca^2+^ influx [18]. We suspect that this dysregulated Ca^2+^ entry likely affects various Ca^2+^ dependent signaling pathways, leading to changes in gene expression, energy metabolism, and mitochondrial homeostasis, ultimately compromising stress resilience (Fig 6H). Consistent with this assumption, RNA-seq analysis of *shk-1* mutants revealed significant transcriptional changes under basal conditions, including decreased expression of *catp-3*, which encodes a cation-transporting ATPase. This family of ATPases, such as Na^+^/K^+^ pumps, drives the net export of positive charges from the cell [21]. Thus, decreased *catp-3* expression is likely to exacerbate the ion flux and gradient defects in *shk-1* mutants; while overexpressing *catp-3* may restore electrochemical gradients by pumping positive charges out of the cell, alleviating cellular stress caused by excessive Ca^2+^ influx in *shk-1* mutants. Additionally, disruptions in ion homeostasis likely divert cellular energy priorities from storage to ion balance maintenance [45]. This imbalance may also impair glycogen synthesis and mobilization pathways [45]. As a consequence, glycogen stores become depleted in the absence of *shk-1* (Fig 6H).

In addition, prolonged Ca^2+^ influx in *shk-1* mutants may cause sustained mitochondrial Ca^2+^ accumulation, similar to what occurs in pathological conditions such as ischemia/reperfusion injury [46,47]. Normally, tightly regulated mitochondrial Ca²⁺ uptake supports ATP production and maintains energy homeostasis. However, excessive Ca²⁺ accumulation in the mitochondria can elicit adaptive and maladaptive responses, depending on the magnitude and duration of the stress [47–49]. We suspect that the mild mitochondrial uncoupling (Fig 5C and 5D) and partial ΔΨm depolarization (Figs 5F and 6G) in *shk-1* mutants possibly reflect protective and adaptive strategies against elevated mitochondrial Ca²⁺ load. These changes help mitigate ROS production and prevent further Ca²⁺ uptake by the mitochondria, resembling protective responses observed in ischemic preconditioning [47]. Moreover, such adaptations may blunt the usual Ca^2+^ sensitivity of depolarizing mechanisms, preventing effective ΔΨm depolarization upon subsequent oxidative challenges [47,50]. While these responses initially enhance cellular resilience, their prolonged persistence often exceeds the cell’s adaptive capacity, causing deleterious consequences [48]. A sustained drop, even mild, in ΔΨm and persistent mitochondrial uncoupling, as observed in *shk-1* mutants (Figs 5C, 5D, 5F and 6G), can lead to chronically reduced ATP production and persistent energy inefficiency. One consequence of these long-lasting maladaptive responses could be the continuous depletion of cellular energy reserves, such as glycogen, resulting in insufficient glucose release to neutralize excessive ROS production and support heightened energy demands under oxidative stress [35,36,48]. Additionally, the inability of *shk-1* mutants to fully depolarize ΔΨm during oxidative stress, due to adaptative mechanisms aimed at reducing stress sensitivity, drives excessive ROS production, as high ΔΨm favors ROS generation [50]. Collectively, these factors accelerate oxidative damage and cellular decline. Another potential explanation for the failed ΔΨm dissipation in *shk-1* mutants involves a mechanism observed in mammals. It has been shown that stress-evoked activation of the proapoptotic Bcl-2 family protein BAX regulates ΔΨm dissipation and ROS production through its interaction with Kv1.3 channels [51]. The BAX-induced changes in ΔΨm and ROS production are abolished in Kv1.3 deficient cells [51]. While speculative, a similar mechanism might explain the failed ΔΨm depolarization in *C. elegans shk-1* mutants, though this requires further investigation.

Interestingly, Kv1.3 channels in mammals are also involved in regulating energy metabolism, suggesting that our findings may have broader implications beyond *C. elegans* [13,14]. Disruption or pharmacological inhibition of Kv1.3 leads to weight loss, enhanced insulin sensitivity, and decreased plasma glucose levels, without affecting food intake or activity [13,14]. Moreover, mice lacking Kv1.3 exhibit resistance to diet-induced obesity [13,14]. These metabolic effects are thought to result from an increased translocation of the glucose transporter GLUT4 to the plasma membrane [14]. Building on these observations, it would be intriguing to determine if the translocation of FDGT-1, the *C. elegans* ortholog of GLUT4, is altered in *shk-1* mutants and if this alteration can be reversed by increased *catp-3* expression. Additionally, investigating if disruption of Kv1.3 in mammals leads to decreased expression of the CATP-3 ortholog, and if increased expression of this ortholog can rescue the GLUT4 translocation defects in Kv1.3-deficient cells, could provide insights into the conserved mechanisms by which Shaker/Kv1 channels regulate energy homeostasis.

In mammals, Kv1.3 is not only localized to the plasma membrane but is also present in other subcellular compartments including mitochondria [52–54]. However, both SHK-1 and CATP-3 in *C. elegans* lack the canonical mitochondrial targeting sequences at their N-terminal domains and have not been detected in the previous mitochondrial proteomic analysis [55]. This implies that SHK-1 and CATP-3 may not act directly within mitochondria to regulate mitochondrial homeostasis in worms, although we cannot entirely rule out the possibility that they play such a role.

In summary, our findings suggest that excessive Ca^2+^ influx and disrupted ion homeostasis likely contribute to the diverse phenotypes observed in *shk-1* mutants (Fig 6H), although direct evidence for this connection is currently lacking. Nevertheless, rescuing the defects in *shk-1* mutants by increasing the expression of the cation-transport ATPase gene *catp-3* implicates a link between ion imbalance and compromised stress tolerance in these animals. Our results may also have broader implications for understanding how mammalian Shaker/Kv1 channels regulate various physiological processes, including energy homeostasis and mitochondrial function.

## Materials and Methods

### *C. elegans* strains

Worms were maintained as previously described [56]. The strains used in this study are listed in S3 Table.

### Constructs and transgenic animals

Expression vectors were generated using Multisite Gateway System (ThermoFisher Scientific). The promoters, including *shk-1* (3.3 kb), *myo-3* (2.5 kb), *rab-3* (1.2 kb), *catp-2* (4.8 kb), *catp-3* (4 kb), *catp-4* (3.8 kb), *catp-6* (3.5 kb), *catp-7* (3.9 kb), *zip-8* (3.8 kb), and *cav-1* (4.1 kb) were amplified from genomic DNA and cloned to pDONR P4P1 using BP reaction. Genes of interest, including *shk-1* cDNA and genomic sequences (*zip-8, cav-1, catp-2, catp-3, catp-4, catp-6,* and *catp-7*) were amplified using either the synthesized fragment (IDT) or genomic DNA as the template. The transgenic animals were generated by injecting expression vectors at a concentration of 50 ng/μl together with 50 ng/μl co-injection marker.

### CRISPR-Cas9 knockout

Genes were disrupted using CRISPR/Cas9-mediated genome editing as described [26]. It involved the homology-directed insertion of a pre-designed single strand DNA template (ssODN), which contains two 35-base homology arms flanking the targeted PAM site and a short sequence containing a unique restriction enzyme cutting site and stop codons in all three frames between two homology arms. In addition, the insertion of ssODN template removes 16 bases of the coding sequence, ensuring the proper gene disruption. The injection cocktail was prepared as follows: 0.5 μl of Cas9 protein (IDT, #1081059) was mixed with 5 μl of 0.4 μg/μl tracrRNA (IDT, #1072534) and 2.8 μl of 0.4 μg/μl crRNA (IDT). The mixture was incubated at 37 °C for 10–15 minutes. Subsequently, 2.2 μl of 1 μg/μl ssODN (or 500 ng dsDNA) and 2 μl of 0.6 μg/μl *rol-*6 co-injection marker were added. The final volume was brought to 20 μl with nuclease-free water. The injection mixture was centrifuged for 5 minutes before use. To disrupt specific isoform(s) of the *shk-1* gene, the unique regions of different isoforms were targeted.

To endogenously tag *shk-1* with GFP, the knock-in template was generated by amplifying the GFP sequence using primers, which contain homology arms flanking the *shk-1* stop codon. The PCR product was purified using AMPure XP beads (A63880, Beckman Coulter), and the injection mix was prepared as previously described [57,58]. Detailed information on the crRNA and ssODN used in this study can be found in S3 Table.

### Bacterial strains

The *V. cholerae* A1552 and *P. aeruginosa* PA14 were grown in LB broth at 37 °C. *E. faecalis* OG1RF and *S. aureus* were grown in brain heart infusion (BHI) broth or in tryptic soy (TS) broth at 37 °C, respectively. To prepare bacterial lawns, a single bacterial colony was inoculated into 2 ml of the corresponding broth mentioned above, and grown on a shaker for 12 hours at 37 °C. For *V. cholerae* A1552, the overnight culture was diluted (1:100) with LB to a volume of 10 ml and grown to OD = 2.0 at 37 °C before 150 µl was seeded on the center of a 5.5 cm NGM plate to generate a pathogen lawn of about 1.5 cm in diameter or to cover the entire plate surface. For other three bacteria, 20 μl of overnight culture was spotted onto the center of a 3.5 cm plate. These plates were incubated at 37 °C for 12–16 hours except for *S. aureus*, which was incubated for 6 hours. The lawns of *E. coli* TOP10 and *E. coli* TOP10/*pmakB*^*+*^*C*^*+*^*D*^*+*^*A*^*+*^ were prepared in the same way as *V. cholerae* A1552. For avoidance assays, synchronized L4 animals fed with OP50 were washed three times with M9, and 30 animals of each strain were used in each assay. The number of animals staying on the bacterial lawn was counted at different time points. For the killing assays, synchronized L4 animals fed with OP50 were washed 3 times in M9, and 30 animals were used in each assay. The killing was scored each day until all worms were dead. Worms were considered dead when they did not respond to the prodding at both head and tail. The number of surviving animals was counted and documented each day. To avoid contamination from their offspring, the animals were transferred to fresh assay plates every second day. The mean survival changes were computed as (mean survival of the mutant – mean survival of wild type)/ mean survival of wild type. Three biological replicates were included for each figure plot except for the screen with *V. cholerae* A1552, in which each mutant was assayed twice. Survival curves were plotted using GraphPad Prism, and log-rank tests were performed to compare statistical differences between different genotypes or treatments.

### Lifespan assays

Lifespan was assayed at room temperature (~22 °C). In brief, 30 synchronized L4 animals were transferred to each assay plate, marking time 0. To avoid contamination by the offspring, animals were transferred to new assay plates with OP50 or heat-killed *V. cholerae* every second day, and the number of live worms was counted daily until all had died. Animals that climbed up the walls of the assay plates and subsequently disappeared were excluded from the analysis. Animals were considered dead if they did not respond to prodding at both head and tail. Survival curves were generated using GraphPad Prism, and log-rank tests were performed to assess statistical differences across various genotypes.

### *V. cholerae*-GFP colonization assay

Bacterial culture was prepared in 2 ml of LB broth with 100 μg/ml ampicillin, following the same protocol used for *V. cholerae* A1552. Subsequently,150 μl of the culture was spread on the surface of 5.5 cm NGM plates. The plates were incubated for 12–16 hours at 37 °C and then cooled to room temperature before seeding with synchronized L4 animals. After 16 hours of exposure, the animals were transferred from the *V. cholerae*-GFP plates to OP50 plates to remove the GFP positive bacteria attached to the skin. The GFP fluorescence was visualized within 5 minutes of cleaning under Nikon A1 confocal microscope.

### Quantification of intestinal bacterial loads

For the quantification of colony-forming unit (CFU) after 16 hours of exposure, bacterial lawns of *V. cholerae*-GFP were prepared by spreading 150 μl of culture on the surface of 5.5 cm NGM agar plates. The plates were incubated for 12–16 hours at 37 °C and cooled to room temperature before synchronized L4 worms were placed on the plates. The assays were performed at room temperature. After exposure, the animals were transferred from *V. cholerae*-GFP plates to the OP50 plates for 10 min to remove *V. cholerae*-GFP from their body surfaces. This cleaning step was conducted three times in total. Subsequently, ten animals per genotype were transferred into 50 μl of PBS supplemented with 0.01% Triton X-100 and ground. Serial dilutions of the lysates (10^1^, 10^2^, 10^3^, 10^4^) were plated onto LB plates containing 100 μg/ml of ampicillin and incubated overnight at 37 °C. In the next day, the number of single colonies was counted and represented as CFU per animal.

### Pumping measurement

The pumping rate was measured by counting the number of pumps in a minute. For each genotype, day-one adult animals were used to count the number of contractions of the terminal bulb. One backward movement of the grinder in the terminal bulb was defined as one contraction. The pumping rates were recorded for 30 animals per genotype.

### Oxidative stress assays

Synchronized L4 worms were picked to NGM plates with OP50 and grown overnight. Thirty day-one adults were transferred to each assay plate in the presence of 7.7 mM sodium arsenite or 75 mM paraquat. The assay plates were freshly made the day before the assay. 50 μl of 10-fold concentrated OP50 was spotted to the plate center 2–3 hours before the assay. The survival was scored every 2 hours until all had died. Animals that climbed up the walls of the assay plate and disappeared were excluded from the analysis. Worms were considered dead when they did not respond to prodding at both head and tail. Three biological replicates, each with three technical replicates, were included for each genotype. Similar to the pathogen experiments, survival curves of oxidative stress assays were generated using GraphPad Prism, and log-rank tests were conducted to assess statistical differences across various genotypes or treatments.

### RNAi by feeding

RNAi plates were prepared by supplementing NGM plates with 1 mM IPTG and 50 μg/ml ampicillin [59]. RNAi-expressing bacteria were spread on these plates, and dsRNA expression was induced overnight at room temperature in a dark room. Ten day-one adults were placed on the induced RNAi plates and allowed to lay eggs for 2 hours. After three days of RNAi, animals were assayed in plates with the presence of 7.7 mM sodium arsenite as described above.

### N-acetylcysteine and mitoTemp treatment

The stock solutions of N-acetylcysteine (NAC) (A7250, Sigma-Aldrich) and mitoTemp (SML0737, Sigma-Aldrich) were prepared at 500 mM in ddH_2_O. NAC or mitoTemp were added to assay plates at final concentrations of 5 mM and 500 μM, respectively. The assay plates were prepared the day before the assay, and 30 day-one adults were used in each assay. Three biological replicates, each with three technical replicates, were included for each genotype.

### Glucose and Trehalose treatment

D-(+)-glucose and D-(+)-trehalose dihydrate were added to NGM plates to achieve final concentration of 2% glucose and 5 mM trehalose, along with 0.1 mg/ml kanamycin. The plates were then seeded with 100 μl of a 10-fold concentrated OP50 and dried for 30 minutes. 10 young adult worms were transferred to the glucose or trehalose plates and allowed to lay eggs for 2 hours. After egg hatching, animals were grown in the presence of glucose or trehalose till day-one adults, which were assayed on the pro-oxidant containing plates.

### TMRE staining

Tetramethylrhodamine ethyl ester (TMRE) staining was performed according to a previously described protocol with minor modifications [60]. TMRE was prepared as a 12.5 mM stock solution in methanol and diluted to 2 μM with M9 buffer before each use. Day-one adult animals were transferred into the staining solution and incubated for 6 hours at room temperature in the dark. After staining, the worms were washed three times in M9 buffer and transferred to a new NGM plate seeded with OP50 for 1 hour in the dark. Worms were subsequently paralyzed using 1 mg/ml levamisole and imaged using a Nikon A1 confocal microscope.

### Iodine staining

Glycogen levels were estimated using iodine staining as previously described [61]. Briefly, day-one adult animals were stained with 1 ml of diluted (1: 15) Lugol’s iodine solution (2% iodine in 4% potassium iodide, Sigma) with shaking for 20 mins at room temperature. Stained worms were washed three times with M9 buffer and mounted immediately for imaging. The bright-field images were captured using a Nikon Eclipse E800 with Nikon DS-Ri1 camera and analyzed in Fiji ImageJ.

### Glycogen and ATP measurement

The assessment of glycogen and ATP levels in worms was performed as previously described [62,63]. Synchronized day-one adults were collected and washed three times with M9 buffer to remove the bacteria. The collected worms were frozen in liquid nitrogen, followed by boiling for 15 min. The samples were centrifuged at 14,000 rpm for 10 min at 4 °C, and the supernatant was transferred to a new 1.5 ml tube for the downstream analysis. Glycogen and ATP levels were determined by using the commercially available kits (ab65620 Abcam for glycogen, and A22066 Molecular Probes for ATP) according to the manufacturer’s protocol. Three biological replicates, each with three (glycogen) or two (ATP) technical replicates, were included for each genotype.

### The measurement of Oxygen Consumption Rate (OCR)

To evaluate changes in mitochondrial activity, O_2_ consumption was measured using the Seahorse XFp system (Agilent Technologies). Seahorse XFp sensor cartridges were hydrated in deionized water for 16 hours and then in XF calibrant for 1 hour in a non-CO_2_ incubator at 37 °C. Synchronized day-one adults were picked into 180 μl of M9 in Seahorse XFp culture plates. Each well contained approximately 10 worms, with at least 3 wells per genotype. Carbonyl cyanide 4-(trifluoromethoxy) phenyl-hydrazone (FCCP, 100 μM, Sigma Aldrich) and sodium azide (400 mM) were added to injection ports A and B of the sensor cartridge, respectively. The MitoStress assay was run at 20 °C using the following parameters: 3 minutes of mixing, 3 minutes of measuring for 4 basal cycles, followed by 4 cycles after FCCP injection and 4 cycles after sodium azide injection. O_2_ consumption rate (OCR) was normalised to the number of worms per well. Data was exported from the Seahorse XFp analyser into Seahorse XF Report Generator software.

### Confocal microscopy

Worms were paralyzed with 50 mM NaN_3_ or 1 mg/ml levamisole, and mounted on glass slides coated with 2% agarose in M9 buffer. The images were acquired under Nikon A1 confocal microscope with Nikon NIS elements software. The images were analyzed using Fiji ImageJ and represented as maximum projection of confocal z-stacks.

### RNA extraction

Twenty young adult animals were picked to the fresh plates and allowed to lay eggs for 2 hours. The adults were then removed, and eggs were allowed to develop into the L4 stage. Ten plates of synchronized L4 animals were collected, washed with M9 buffer, and placed on either OP50 or *V. cholerae* A1552 plates for 8 hours. After exposure, the animals were washed off and rinsed three times with M9 buffer. The worm pellets were then homogenized using Bullet Blender (Next Advance) in the presence of Qiazol Lysis Reagent and 0.5 mm Zirconia beads at 4 °C. RNA was extracted using the RNeasy Plus Universal Mini Kit (Qiagen) according to the kit instruction. For both OP50 and *V. cholerae* exposure, five independent RNA samples were prepared, and libraries were constructed and sequenced by Novogene.

### RNA-seq analysis

RNA samples of wild type and *shk-1* mutants were prepared and sequenced in parallel under the identical conditions and treatment. The RNA-seq of wild type, which has been used earlier [26], served as a control. The raw reads were aligned to the *C. elegans* WBcel235.103 reference genome using STAR v2.7.9a [64]. The genome index was built using additional parameters --sjdbOverhang 150 --genomeSAindexNbases 10. Counts per gene were computed using featureCounts [65]. The data was then analyzed in R using DESeq2 v1.36.0 for differential expression [66]. GO analysis was performed using enrichR v3.2 [67]. The GO of WT samples [26] were re-analyzed with modified R code to enable comparison with that of *shk-1* mutants. The positive and negative fold genes were analyzed separately. Plotting was done using ggplot and ggVennDiagram.

## Supporting information

S1 Fig Screening for K^+^ channel mutants with altered susceptibility to *V. cholerae.* **(A–F)** Survival of K^+^ channel mutants upon exposure to *V. cholerae* A1552. In the screen, each mutant was assayed twice. **(G)** Survival of WT, *shk-1(yum1003)*, *shl-1(yum1098)* and *shk-1(yum5132); shl-1(yum5133)* animals, upon exposure to *V. cholerae* A1552. n = 3 biological replicates. *p* values are displayed in the plot. log-rank test. **(H)** Survival of WT and *shk-1(yum1003)* upon exposure to heat-killed *V. cholerae* A1552. n = 3 biological replicates. *p* value is displayed in the plot. log-rank test. **(I)** The percentage of WT and *shk-1(yum1003)* remaining on *E. coli* Top10 lawn with or without the expression of *makD/C/B/A* operon at various time points. n = 3 biological replicates. ns = not significant. Two-tailed *t* test. **(J)** Survival of WT and *shk-1(yum1003)* animals upon exposure to *E. coli* Top10 stain with or without the expression of *makD/C/B/A* operon. n = 3 biological replicates. *p* values are displayed in the plot.
(TIF)

S2 Fig V. cholerae infection elicits robust defense responses in wild type and shk-1 mutants. **(A–B)** GO categories for down- (A) and up- (B) regulated genes following 8 hours of *V. cholerae* A1552 infection in wild type and *shk-1(yum1003)* mutant animals with adjusted *p* <1e-20. **(C)** Scatter plot showing the expression fold changes for differentially expressed genes within the GO category ‘stress response’ with adjusted *p* <1e-20 in wild type and *shk-1(yum1003)* mutants, following *V. cholerae* A1552 infection.
(TIF)

S3 Fig SHK-1 is required for oxidative stress response. **(A and B)** Survival of WT and *shk-1* mutants upon exposure to 7.7 mM arsenite (A) or 75 mM paraquat (B). #1*(yum1003)* and #2 *(yum1018)* indicate two independent null alleles of *shk-1*. n = 3 biological replicates. *p* values are displayed in the plots. log-rank test. **(C)** Survival of WT, *shk-1(yum1003), skn-1(lax120) and shk-1(yum1003); skn-1(lax120)* upon exposure to 7.7 mM arsenite. *lax120* is a gain-of-function allele of *skn-1*. n = 3 biological replicates. *p* values are displayed in the plot. log-rank test. **(D)** Survival of WT, *shk-1(yum1003), skn-1(lax188) and shk-1(yum1003); skn-1(lax188)* upon exposure to 7.7 mM arsenite. *lax188* is a gain-of-function allele of *skn-1*. n = 3 biological replicates. *p* values are displayed in the plot. log-rank test. **(E)** Schematic drawing of all *shk-1* isoforms. The arrows indicate the CRISPR/Cas9 targeting sites. **(F)** Survival of WT, *shk-1(yum1003, disrupting all isoforms), shk-1(yum 1012, disrupting isoforms c and d), shk-1 (yum 1013, disrupting isoform e), shk-1(yum 1014, disrupting isoforms b, c, and d)*, and *shk-1 (yum 1015*, *disrupting isoforms a, b, and c).* The CRISPR/Cas9 targeting sites are displayed in (E). n = 3 biological replicates. *p* values are displayed in the plot. log-rank test. **(G)** Representative DIC and fluorescent images showing the endogenous SHK-1-GFP expression. The arrow indicates the IL2L neuron. **(H)** Survival of WT, *shk-1(yum1003),* transgenic *shk-1(yum1003)* expressing *shk-1* cDNA under *myo-3* or *rab-3* promoter upon exposure to *V. cholerae*. n = 3 biological replicates. *p* values are displayed in the plot. log-rank test. **(I)** Survival of *rde-1(ne300)* mutants and animals treated with RNAi against *shk-1* in the *rde-1(ne300)* background, both with and without restored RNAi capacity in the body wall muscle, through the expression of wild-type *rde-1* under the control of *hlh-1* promoter. n = 3 biological replicates. *p* values are displayed in the plot. log-rank test. **(J)** Survival of WT, *shk-1(yum1003), flcn-1(yum1020)*, and *shk-1(yum1003); flcn-1(yum1020)* animals upon exposure to *V. cholerae*. n = 3 biological replicates. *p* values are displayed in the plot. log-rank test.
(TIF)

S4 Fig Altered expression of *zip-8* and *cav-1* does not affect oxidative stress response in *shk-1* mutants. **(A)** Volcano plot highlighting the expression of *zip-8* and *cav-1* genes in *shk-1* (*yum1003*) mutants relative to WT. **(B)** Survival of WT, *shk-1(yum1003), zip-8* RNAi, and *shk-1(yum1003)*; *zip-8* RNAi animals upon exposure to 7.7 mM arsenite. n = 3 biological replicates. *p* values are displayed in the plot. log-rank test. **(C)** Survival of WT, *shk-1(yum1003*), *cav-1(yum1024)*, and *shk-1(yum1003); cav-1(yum1024)* double mutants upon exposure to 7.7 mM arsenite. n = 3 biological replicates. *p* values are displayed in the plot. log-rank test. **(D and E)** Survival of WT and animals with *zip-8* overexpression (OE) (D), or WT and animals with *cav-1* overexpression (OE) (E) upon exposure to 7.7 mM arsenite. n = 3 biological replicates. *p* values are displayed in the plots. log-rank test.
(TIF)

S5 Fig The survey of *catp* genes in regulating SHK-1 mediated oxidative stress response. **(A–E)** Survival of WT and *catp-2(yum1091)* (A), WT and *catp-3(yum1092)* (B), WT and *catp-4(yum1093)* (C), WT and *catp-6(yum2822)* (D), and WT and *catp-7(yum2823)* (E) upon exposure to 7.7 mM arsenite. n = 3 biological replicates. *p* values are displayed in the plots. log-rank test. **(F–I)** Survival of WT and *shk-1(yum1003)*, with or without the overexpression of *catp-2* genomic DNA (F), *catp-4* genomic DNA (G), *catp-6* genomic DNA (H), and *catp-7* genomic DNA (I) under their endogenous promoters, upon exposure to 7.7 mM arsenite. n = 3 biological replicates. *p* values are displayed in the plots. log-rank test. **(J)** Survival of WT and *catp-3(yum1092)* animals upon exposure to *V. cholerae* A1552. n = 3 biological replicates. *p* value is displayed in the plot. log-rank test. **(K and L)** Survival of WT and *shk-1(yum1003)*, with or without the overexpression of *catp-3* genomic DNA under either its endogenous promoter (K) or a body-wall muscle specific promoter (L), upon exposure to *V. cholerae* A1552.
(TIF)

S1 Table Systematic screening of K+ channels mutants for susceptibility to *V. cholerae* infection. Each K^+^ channel mutant was assayed twice in response to *V. cholerae* exposure. The mean survival of each mutant was compared to that of WT. The numbers displayed in the table represent the mean survival changes of mutant animals upon *V. cholerae* exposure. This was calculated as (mean survival of the mutant – mean survival of wild type)/ mean survival of wild type.
(XLSX)

S2 Table RNA-seq analysis of gene expression in WT and *shk-1* mutants following 8-hour infection with *V. cholerae.* **(A)** The log2 fold change of gene expression in *shk-1* mutants relative to that of WT animals without *V. cholerae* infection (uninf). **(B)** The log2 fold change of gene expression in WT animals following 8 hours of *V. cholerae* infection (inf), compared to WT animals without infection (uninf). **(C)** The log2 fold change of gene expression in *shk-1* mutants following 8 hours of *V. cholerae* infection (inf), compared to *shk-1* mutants without infection (uninf).
(XLSX)

S3 Table Strains and reagents used in this study. (XLSX)

S1 Data Underlying data for figures. (XLSX)
