# Supplementary figures and images for: Shaker/Kv1 potassium channel SHK-1 protects against pathogen infection and oxidative stress in C. elegans
Source: PLoS Genet. 2025 Feb 6;21(2):e1011554. doi: 10.1371/journal.pgen.1011554 (PMC11849984; doi:10.1371/journal.pgen.1011554)

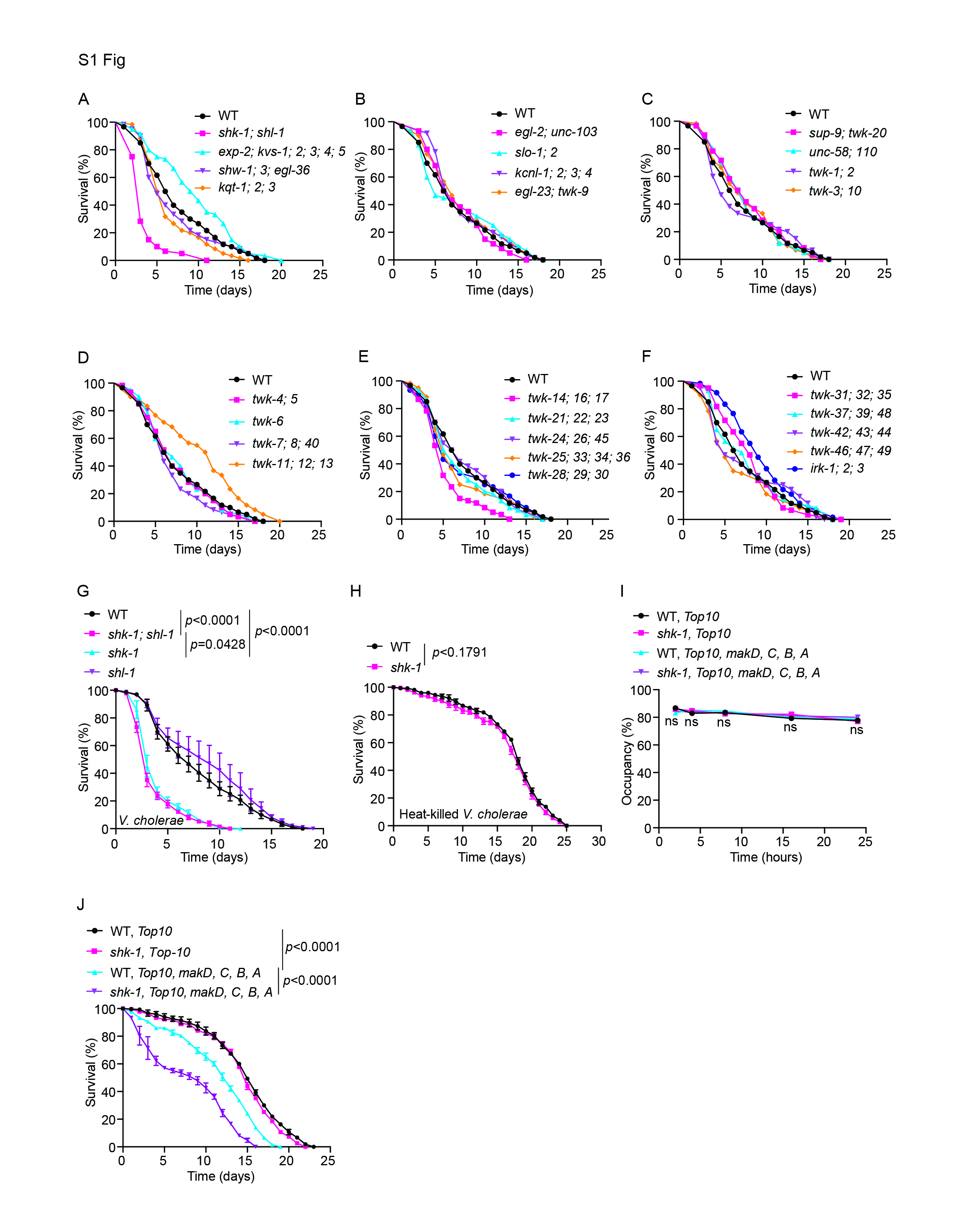

Supplement: S1 Fig — (A–F) Survival of K+ channel mutants upon exposure to V. cholerae A1552. In the screen, each mutant was assayed twice. (G) Survival of WT, shk-1(yum1003), shl-1(yum1098) and shk-1(yum5132); shl-1(yum5133) animals, upon exposure to V. cholerae A1552. n = 3 biological replicates. p values are displayed in the plot. log-rank test. (H) Survival of WT and shk-1(yum1003) upon exposure to heat-killed V. cholerae A1552. n = 3 biological replicates. p value is displayed in the plot. log-rank test. (I) The percentage of WT and shk-1(yum1003) remaining on E. coli Top10 lawn with or without the expression of makD/C/B/A operon at various time points. n = 3 biological replicates. ns = not significant. Two-tailed t test. (J) Survival of WT and shk-1(yum1003) animals upon exposure to E. coli Top10 stain with or without the expression of makD/C/B/A operon. n = 3 biological replicates. p values are displayed in the plot. (TIF) [file pgen.1011554.s001.tif]

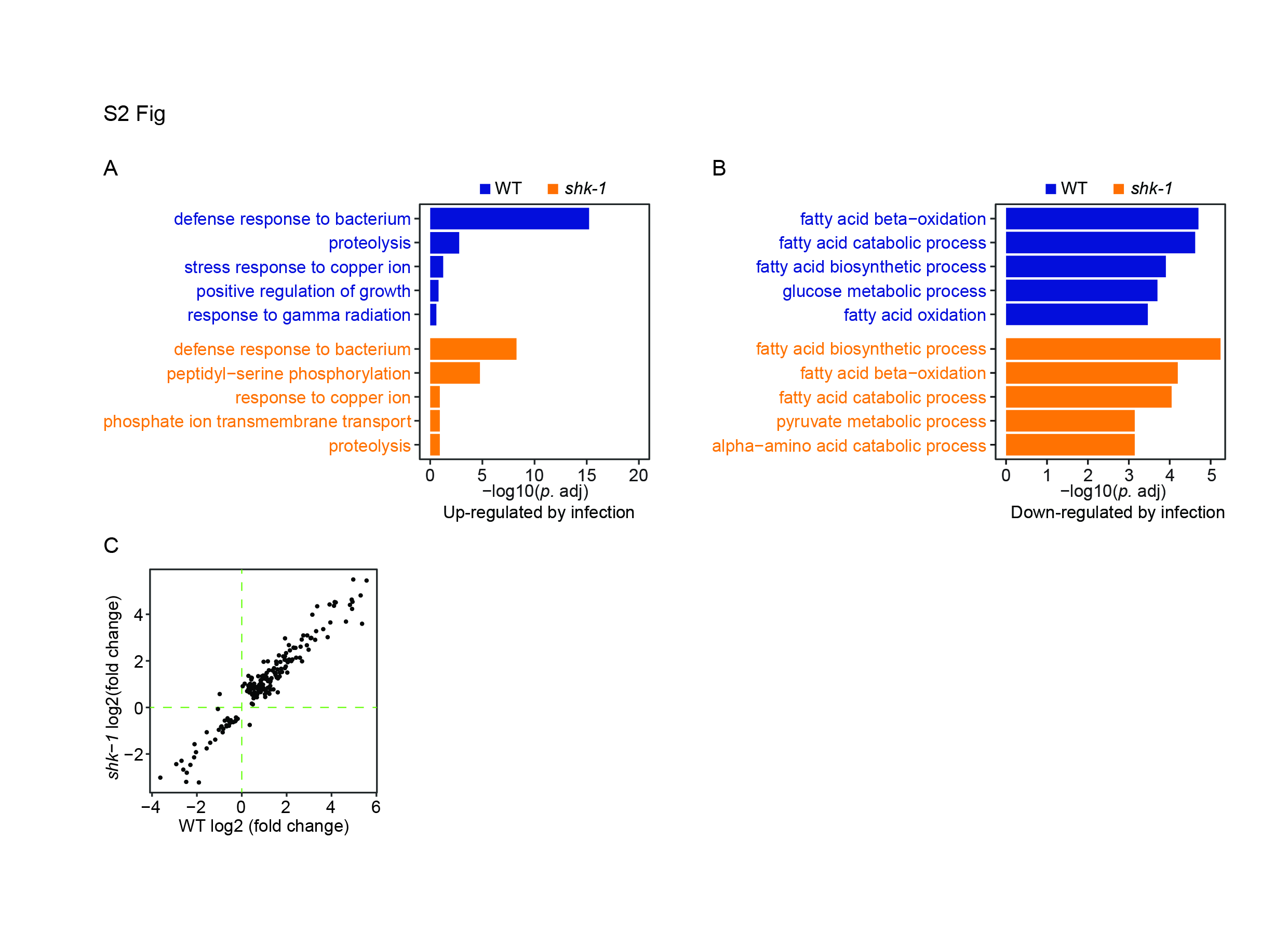

Supplement: S2 Fig — (A–B) GO categories for down- (A) and up- (B) regulated genes following 8 hours of V. cholerae A1552 infection in wild type and shk-1(yum1003) mutant animals with adjusted p <1e-20. (C) Scatter plot showing the expression fold changes for differentially expressed genes within the GO category ‘stress response’ with adjusted p <1e-20 in wild type and shk-1(yum1003) mutants, following V. cholerae A1552 infection. (TIF) [file pgen.1011554.s002.tif]

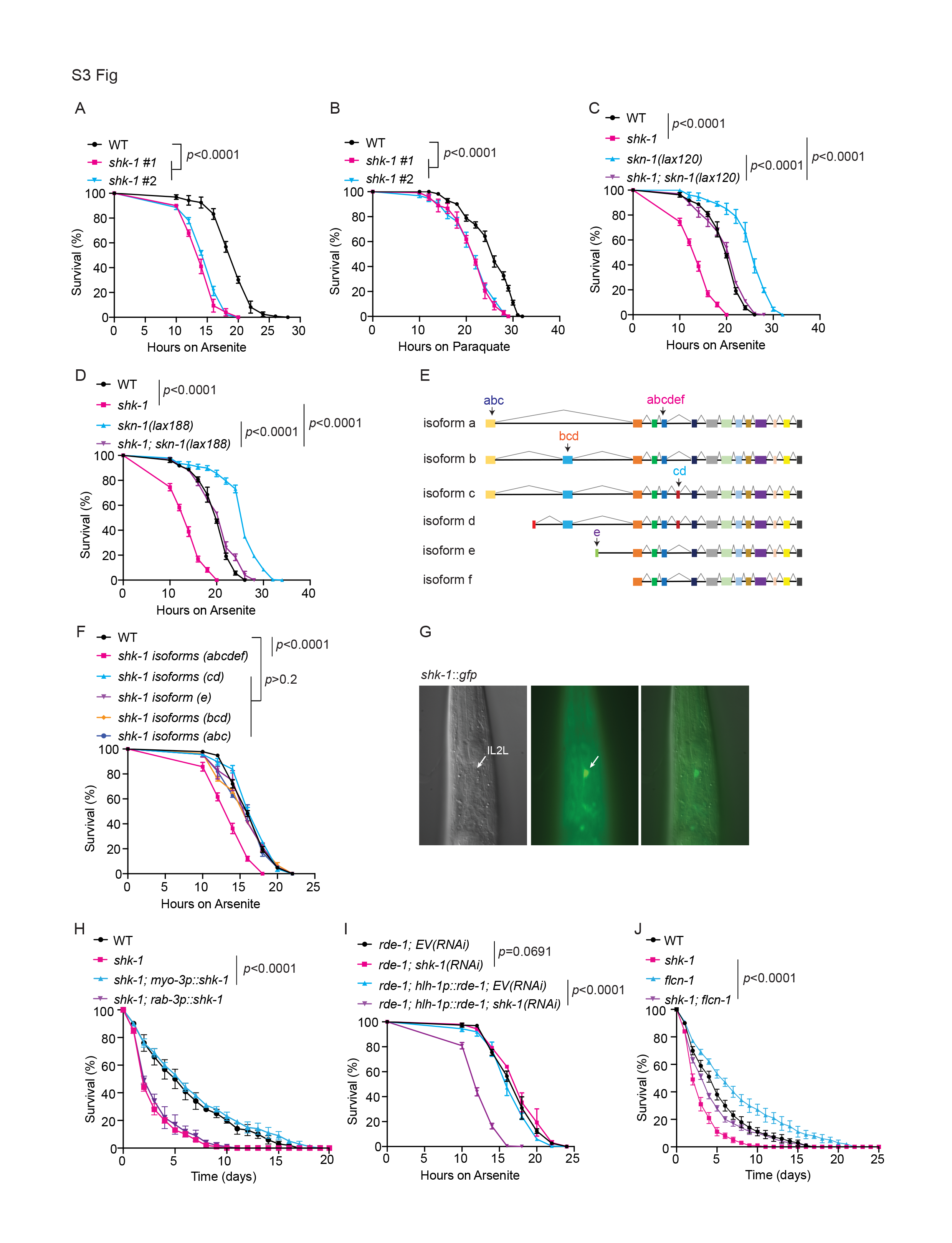

Supplement: S3 Fig — (A and B) Survival of WT and shk-1 mutants upon exposure to 7.7 mM arsenite (A) or 75 mM paraquat (B). #1(yum1003) and #2 (yum1018) indicate two independent null alleles of shk-1. n = 3 biological replicates. p values are displayed in the plots. log-rank test. (C) Survival of WT, shk-1(yum1003), skn-1(lax120) and shk-1(yum1003); skn-1(lax120) upon exposure to 7.7 mM arsenite. lax120 is a gain-of-function allele of skn-1. n = 3 biological replicates. p values are displayed in the plot. log-rank test. (D) Survival of WT, shk-1(yum1003), skn-1(lax188) and shk-1(yum1003); skn-1(lax188) upon exposure to 7.7 mM arsenite. lax188 is a gain-of-function allele of skn-1. n = 3 biological replicates. p values are displayed in the plot. log-rank test. (E) Schematic drawing of all shk-1 isoforms. The arrows indicate the CRISPR/Cas9 targeting sites. (F) Survival of WT, shk-1(yum1003, disrupting all isoforms), shk-1(yum 1012, disrupting isoforms c and d), shk-1 (yum 1013, disrupting isoform e), shk-1(yum 1014, disrupting isoforms b, c, and d), and shk-1 (yum 1015, disrupting isoforms a, b, and c). The CRISPR/Cas9 targeting sites are displayed in (E). n = 3 biological replicates. p values are displayed in the plot. log-rank test. (G) Representative DIC and fluorescent images showing the endogenous SHK-1-GFP expression. The arrow indicates the IL2L neuron. (H) Survival of WT, shk-1(yum1003), transgenic shk-1(yum1003) expressing shk-1 cDNA under myo-3 or rab-3 promoter upon exposure to V. cholerae. n = 3 biological replicates. p values are displayed in the plot. log-rank test. (I) Survival of rde-1(ne300) mutants and animals treated with RNAi against shk-1 in the rde-1(ne300) background, both with and without restored RNAi capacity in the body wall muscle, through the expression of wild-type rde-1 under the control of hlh-1 promoter. n = 3 biological replicates. p values are displayed in the plot. log-rank test. (J) Survival of WT, shk-1(yum1003), flcn-1(yum1020), and shk-1(yum1003); [file pgen.1011554.s003.tif]

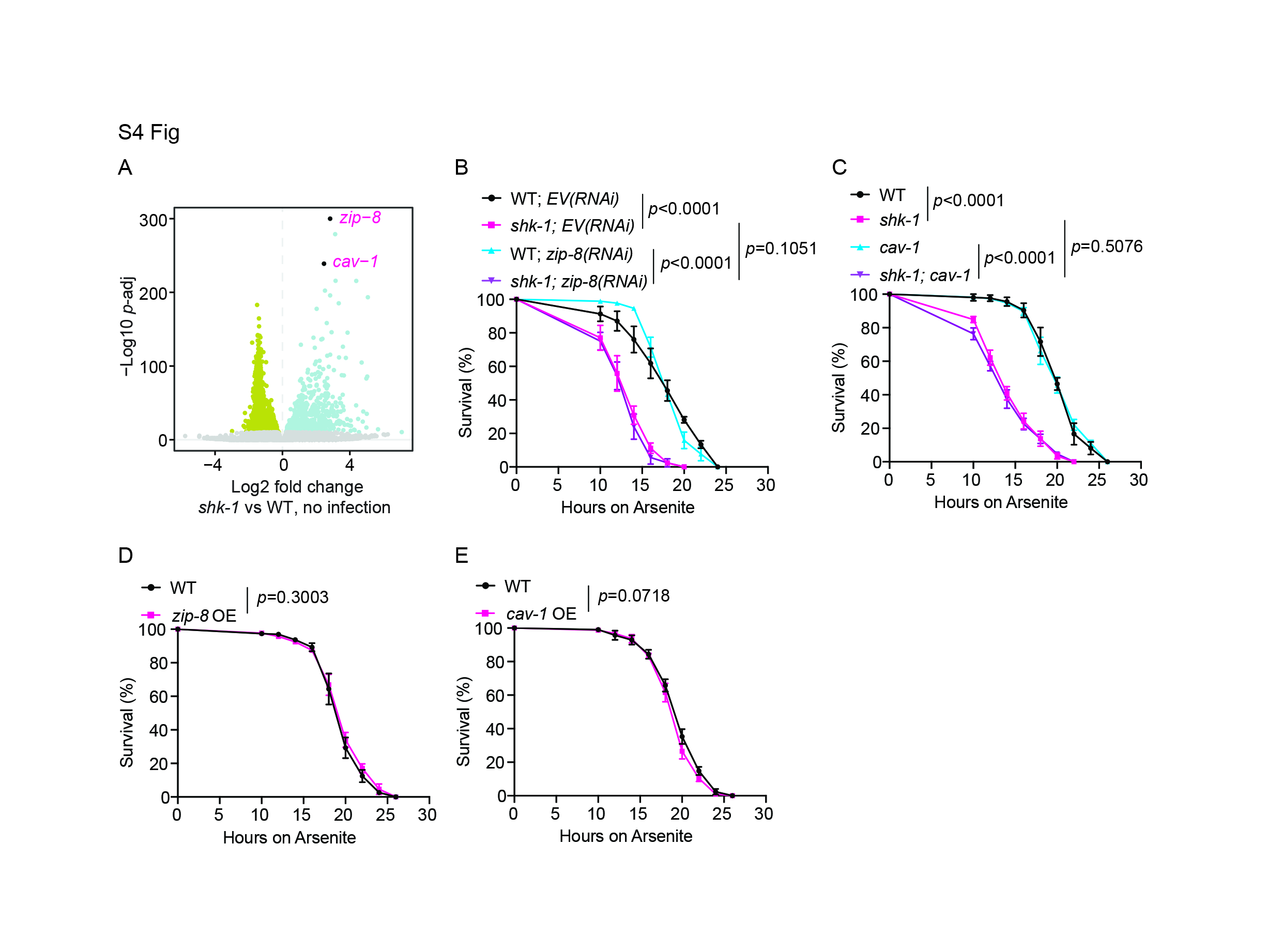

Supplement: S4 Fig — (A) Volcano plot highlighting the expression of zip-8 and cav-1 genes in shk-1 (yum1003) mutants relative to WT. (B) Survival of WT, shk-1(yum1003), zip-8 RNAi, and shk-1(yum1003); zip-8 RNAi animals upon exposure to 7.7 mM arsenite. n = 3 biological replicates. p values are displayed in the plot. log-rank test. (C) Survival of WT, shk-1(yum1003), cav-1(yum1024), and shk-1(yum1003); cav-1(yum1024) double mutants upon exposure to 7.7 mM arsenite. n = 3 biological replicates. p values are displayed in the plot. log-rank test. (D and E) Survival of WT and animals with zip-8 overexpression (OE) (D), or WT and animals with cav-1 overexpression (OE) (E) upon exposure to 7.7 mM arsenite. n = 3 biological replicates. p values are displayed in the plots. log-rank test. (TIF) [file pgen.1011554.s004.tif]

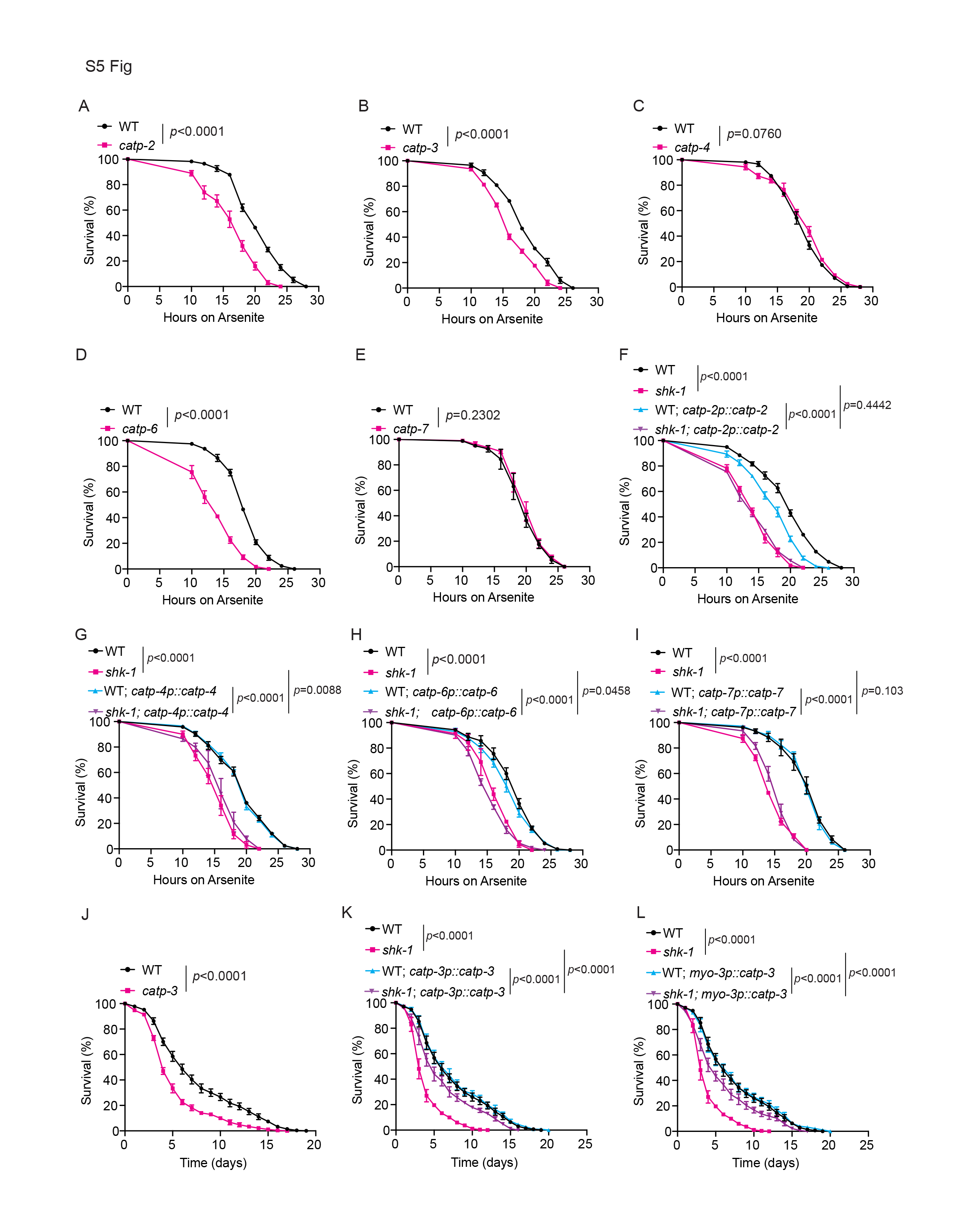

Supplement: S5 Fig — (A–E) Survival of WT and catp-2(yum1091) (A), WT and catp-3(yum1092) (B), WT and catp-4(yum1093) (C), WT and catp-6(yum2822) (D), and WT and catp-7(yum2823) (E) upon exposure to 7.7 mM arsenite. n = 3 biological replicates. p values are displayed in the plots. log-rank test. (F–I) Survival of WT and shk-1(yum1003), with or without the overexpression of catp-2 genomic DNA (F), catp-4 genomic DNA (G), catp-6 genomic DNA (H), and catp-7 genomic DNA (I) under their endogenous promoters, upon exposure to 7.7 mM arsenite. n = 3 biological replicates. p values are displayed in the plots. log-rank test. (J) Survival of WT and catp-3(yum1092) animals upon exposure to V. cholerae A1552. n = 3 biological replicates. p value is displayed in the plot. log-rank test. (K and L) Survival of WT and shk-1(yum1003), with or without the overexpression of catp-3 genomic DNA under either its endogenous promoter (K) or a body-wall muscle specific promoter (L), upon exposure to V. cholerae A1552. (TIF) [file pgen.1011554.s005.tif]
